# Development and Implementation of an Interprofessional, Age-Friendly Learning Academy

**DOI:** 10.1093/geroni/igaf122.2943

**Published:** 2025-12-31

**Authors:** Kristin Zimmerman, Erika Nixon-Lambert, Elvin Price, Lana Sargent, Antara Sarkar, Thomas Walker, Glenda Watkins, Sarah Marrs

**Affiliations:** Virginia Commonwealth University School of Pharmacy, Richmond, Virginia, United States; Virginia Commonwealth University School of Pharmacy, Richmond, Virginia, United States; Virginia Commonwealth University School of Pharmacy, Richmond, Virginia, United States; Virginia Commonwealth University School of Nursing, Richmond, Virginia, United States; Virginia Commonwealth University School of Medicine, Richmond, Virginia, United States; Virginia Commonwealth University School of Medicine, Richmond, Virginia, United States; Virginia Commonwealth University College of Health Professions, Richmond, Virginia, United States; Virginia Commonwealth University College of Health Professions, Richmond, Virginia, United States

## Abstract

There is a shortage of interprofessional geriatrics clinical training and education experiences for health professions students. To address this, we developed an eight-week longitudinal clinical experience for interprofessional learners, including pharmacy, medicine, nursing, social work, physical therapy, and occupational therapy students. The program features co-learning in classroom and clinical spaces, and includes a learner-led design of a quality improvement plan to encourage team-based, age-friendly learning that may translate to collaborative, innovative, person-centered, and continuously improving care. The program was developed by an interprofessional team of faculty and students from the representative disciplines. The development team was guided by the ADDIE (analyze, design, develop, implement, evaluate) model for instructional design. In the analyze phase, the team sought input from faculty and students. In the design phase, the team created learning objectives from key competencies. In the development phase, the four stages of experiential learning from Kolb’s experiential learning cycle (concrete experience, reflective observation, abstract conceptualization, and active experimentation) was used to construct the curriculum. The initial implementation featured iterative feedback from the learner cohort; feedback was incorporated with predetermined learning outcomes and evaluation metrics during the evaluation phase. This presentation will discuss program development and share feedback and learning outcomes from the initial implementation cohort. Specifically, we will share quantitative outcomes related to changes in knowledge of age-friendly care, confidence in providing age-friendly care, interprofessional collaborative competency, dementia comfort and knowledge as well as qualitative outcomes related to intent to change practice and commitment to a career caring for older adults.

